# A Talk on Materia Medica

**Published:** 1896-10

**Authors:** E. C. Briggs

**Affiliations:** Boston Mass.


THE
AMERICAN JOURNAL
—OF—
DENTAL SCIENCE.

VOL. XXX. BALTIMORE, OCTOBER, 1896. NO. 6.

ARTICLE I.

### A TALK ON MATERIA MEDICA.

BY E. C BRIGGS, BOSTON MASS.

Read before the American Academy of Dental Science, December, 27, 1895.

I am perfectly aware that a short time ago I read a paper on "The Dentist as a Prescriber of Drugs," in which I am opposed to the dentist going very much into the constitutional treatment of his patients. At the same time we do have to use drugs, not only locally, but at times systemically. I will begin with the first thing that I have jotted down, and that is, the preparation which I give patients as a soothing application. It is a mixture of equal parts of chloroform, laudnum, and tincture of camphor. That preparation is a stimulant and a slight anesthetic.

A patient will come into your office complaining of some little trouble, some irritation or soreness of the gum, or some inflammation which it would be unwise to attempt to treat at the time, and for such cases I keep this mixture put up in a little vial; and when I think it is needed I tell the office girl to give the patient a bottle of "No. 3," as we call it. Now, that serves a double purpose: It is really very soothing in its effect, and it gives the patient something to do. That is one of the points I want to call your attention to—the importance of doing something for the patient at such times when they are not quite ready for surgical treatment; when you are waiting, perhaps for something to declare itself so that you can make a more clear diagnosis.

In cases where you have filled a root and the patient is threatened with some pain about the root of the tooth, perhaps periostitis or periodontitis, or pericementitis, I have found it necessary in many of those cases to prescribe for the patient some analgesic. In many of these, where it is impossible to do anything surgically, I find that I can do a great deal for patients by giving them some medicine; and of the analgesics which have proved, to be not only safe in my hands, but of really great value are the recent antipyretics that have been discovered,—the several petroleum preparations, acetanilide, phenacetin, etc. One of them, which I have used with marked success, is a proprietary preparation, called "antikamnia." If any of you have never used it, I would recommend you trying it. It is an antipyretic and analgesic, and is composed of acetanilide, with bicarbonate of soda to render it more agreeable to the stomach, and caffeine, the tendency of which is to overcome any depressing effect which the acetanilide might have on the heart. The average dose is three grains, and four of these doses you will find will relieve and stop pain about the facial nerves; such, for instance, as toothache, periostitis; and do it far better than any amount of morphine, and will leave the patient in good condition for the next day. I have found that in giving morphine for toothache the patient must have so much of it to overcome this local pain as to be saturated with it, and to be made ill by it, and it takes a week sometimes to get over the effect, and in the majority of cases the pain has been but indifferently relieved by the drug.

Another drug that is very valuable to us is phenacetin, which is closely allied to the acetanilid which I spoke of, and they are both derived from phenol. This is also safe and very reliable in pains of the kind mentioned, and an excellent form in which to administer it is to give it with citrate of caffeine.

The use of the hypodermic syringe is something that we are becoming more and more familiar with, and we must use it a great deal more in the future. Every man ought to familiarize himself and be accustomed to the use of the hypodermic syringe, so that he may be prepared for cases of collapse and other accidental conditions which may arise. I had a case a little while ago where a patient collapsed from shock, and associated with it was a tobacco heart, and he really seemed to be about to expire. It was a comfort to feel that with the hypodermic syringe I was able to inject brandy under his skin, for he was beyone the ability to swallow. One should be ready in such a case to inject brandy and water, or ammonia, as a heart stimulant; also atropine, and possibly nitro-glycerin. It is a great feeling of satisfaction to know that you were ready when the emergency presented.

The heart can also be reached by the use of nitrite of amyl. Sometimes you get cases of collapse, syncope, when you are using cocaine. I do not think that we often get collapse by cocaine-poisoning, I think it is almost always shock; but, of course, in that case you have to recognize that it may be possible that the cocaine is poisoning the patient, and the nitrite of amyl is a very excellent antidote for cocaine-poisoning. It is bought in these little glass capsules, and it is no skill or effort to prescribe one or to use one,—you simply break it in a napkin and let the patient breathe it. It dilates the capillaries immediately and relieves congestion of the heart, and the circulation is re-established. Right in this connection it is well to speak again of the cases of tobacco heart, and I think we are getting a great many of them. The patient who is afflicted with tobacco heart is just as likely to die in your office as he is to die anywhere, and if there is a little shock, a sudden violent pain, he collapses, if he were somewhere else he probably would die when such a shock occurred. It is highly important that we should be ready to meet such an emergency, should it happen in our office, as it would be very embarrassing and distressing to have him die there.

Then the question of local anesthesia is an important one,—the injection of weak solutions of cocaine submucously, subcutaneously, under the gum and skin. It is one of the greatest helps in practice. Take, for instance, the cases of pyorrhea which need a very heroic treatment, and where, if the patient be not insensible to pain, in nine cases out of ten you will not do the treatment properly—you cannot do it. Then in cases of opening into the antrum and in the many cases of abscess, local anesthesia is of great assistance. Not long ago a patient came to me who had been suffering, walking the floor for three days and nights, with inflammation about the lateral. His dentist had told him he could do nothing for him, as he could not find the cause of it. I opened into the pulp-chamber and found it perfectly clear and free, but the man was suffering and the tooth was so sore and lame it could hardly be touched. Now, the injection of a little cocaine about the root of the tooth enabled me to drill through the foramen and to relieve him immediately of this terrible pain, which was caused by a very slowly forming abscess. It ought to have been formed a couple of days before, but it had not, and there was no swelling to indicate that an abscess was forming. One, of course, could cite innumerable cases where pain has been relieved in this way. The solution of cocain that I use is made from Schleich's formula—viz:

| | | |
|---|---|---|
| R. | Muriate of cocaine . | 20 centigrammes. |
| | Muriate of morphia . . | 25 milligrammes. |
| | Celoride of sodium . | 20 centigrammes. |
| | Water . . . | 100 grammes. |

With a couple of drops of a 1 to 100 solution of carbolic acid. You can even double the cocaine and still be very safe. Supposing you double it, it is only 4-10 of one per cent, and yet that solution, if injected, will enable you to work with great freedom. In the selection of a hypodermic syringe it is very important to get a very, very fine needle. That is where a great many men are intimidated and fail, and think that the use of the drug is as bad as the pain, and it is if you are going to punch in a great clumsy needle; but you can get fine needles, which can be inserted with almost no pain.

Then this question of antiseptics is one which is agitating us all the time. I think we are getting over it a little; we are recovering from the germicide fever,—that idea that we have to put something into every case that is strong enough to kill every germ. I think that is struggling too much for one particular point. We must keep in mind that for the process of putrefaction there must be certain conditions: the germ must be present, and it is necessary to have air and the right condition of heat and of moisture. Now, if you take any of those away you will stop the trouble. The devotion of our entire attention to simply one element of the ferment, the germ, has been carried almost too far,—so far that the reaction has set in to the extent that some men profess not to use any antiseptics. This is fully as irrational as the use of the very powerful germicides. If we can in our operations employ proper antiseptics, mild ones, non-poisonous to the general system, why there can be no harm in making assurance doubly sure, but you must remember that if you have not a good bed for a germ it is not going to get in there.

One of the new drugs which you have heard of, and probably most of you have used, I thought I would speak about, because I have found it so very useful myself, and that is, trichloracetic acid. When acetic acid is acted upon by chlorine, it yields three different combinations: the monochloracetic acid, the dichloracetic acid and the trichloracetic acid; and this trichloracetic acid was discovered to have some special action as a tonic, astringent, and stimulant to the mucous surfaces, and it is also claimed—and I think with truth—to be decidedly effective in making it more easy to remove dental calculi, and it seems to me that in actual practice it has proved to be so; at any rate, it is very excellent in its tonic and stimulant effect on mucous surfaces. The nasal specialist has found it to be of value in the treatment of some of his most obstinate cases, and it is used in antral troubles, in different solutions, according to the case.

The uses of bicarbonate of soda I do not know that all of you appreciate. It is a very simple thing, and one of the beauties of it is that it is so simple and safe that it can be put in the hands of the majority of patients without fear of it doing any harm. Obstinate cases of inflammation about the gum, with puffy, swollen membrane, will yield to this treatment, and the inflammation will subside. I have found it to do good work when other things have failed. It is used with very great benefit in cases of sensitiveness about the necks of the teeth. I have always been a great believer in bicarbonate of soda, and I like to commend it particularly to those who have not used it in the treatment of such cases as I have described.

A word or two about general anesthetics. Of course, not all of us use anesthetics, but still the progress in the use of the different agents for producing anesthesia has been marked of late years, and the subject is of interest to us all. The importance of true anesthesia is to be borne in mind, and that you do not want to carry it beyond that point. If you give an unlimited amount of any anesthetic to a person and carry it to narcotism, you are doing only what you would do with any poisoning drug when you go beyond the prescribed dose. If you were administering opium and morphine, you would consider it extremely dangerous to give more than the maximum dose, and yet in the administration of anesthetics an unlimited amount is provided without much thought of the true therapeutics, the true point to which we wish to carry the patient—that is, anesthesia without carrying it into narcotism.

Right here it is well to speak of the relations of chloroform and ether. Previous to the investigations of the Anglo-Indian Commission at the Hyderabad, it was supposed that chloroform stcpped the action of the heart first, and that ether was safer because it stopped the respiration first. This commission, of which Dr. Lauder Brunton was a member, found that chloroform acted on the respiration first, just as ether does; the only thing is that it is a more delicate drug; it does not take such a dose to produce the narcotic state, and, therefore, it has to be used more carefully.

Men who practice in a specialty of medicine ought to understand how to use in a proper way drugs which it is necessary for them to use in their specialty. I believe that later on we shall use chloroform a great deal more than we do now, and it would simplify matters a great deal, when there is a question as to what anesthetic we should use, if we better understood the qualities and effects of chloroform. Statistics show that in only .0008 of one per cent of cases where anesthetics are administered are there deaths. Of those, if we weed out the ones that are produced by careless overdosing, the ones who died from shock (which might have occurred from any shock), we have an exceedingly small percentage which we can apparently charge to anesthetics themselves. Many of the fatalities which are credited to anesthetics would, if investigated, be found due to other causes. One man in the autopsy was found to have had an abscess in the brain; others have been found far gone in kidney trouble and heart-disease,—and it simply was an accident that they should have died at that particular time. They were likely to die any minute, and as a matter of fact they died easily under an anesthetic.

Dr. Keep, of Springfield, has lately made some researches in the use of anesthetics, and he speaks very strongly about the ability to use chloroform if one uses it rightly, and he gives the dose of chloroform to be half an ounce at the outside. He sometimes combines it with ether, in which case he gives an ounce and a half of ether, but his dose of chloroform is only two drachms. He says that when properly administered complete anesthesia can be obtained, and he fails to see how anybody who has not either one of these severe troubles, kidney, brain, or heart, could possibly suffer from that amount.

Dr. Louis Sayre, of New York, has used chloroform for years in five- to twenty-drop doses, and he has repeatedly asserted it was perfectly safe to use. His method is to give doses from five to twenty drops and to exclude all air, except what was necessary to force the chloroform, and, carrying the patient quickly under this influence, performs his operation without causing the patient any pain.—*International Dental Journal.*

